# The effect of response modality on witness statements when using the self-administered interview

**DOI:** 10.1080/13218719.2024.2313977

**Published:** 2024-04-07

**Authors:** João P. Gomes, Delfina Fernandes, Rui M. Paulo, Pedro B. Albuquerque

**Affiliations:** a School of Psychology, University of Minho, Braga, Portugal; b School of Psychology, Liverpool John Moores University, Liverpool, UK

**Keywords:** digital, eyewitness memory, eyewitness testimony, immediate recall, post-event information, response modality, self-administered interview, witness statement

## Abstract

The Self-Administered Interview (SAI^©^) elicits comprehensive initial statements from witnesses and can enhance subsequent statements. However, the SAI^©^ requires a written response that may have disadvantages compared to a spoken account. This study tested the effect of SAI^©^’s response modality and its subsequent impact on a delayed retrieval attempt. After watching a mock crime, participants completed a Spoken-SAI^©^, Typed-SAI^©^ or no-SAI^©^. Four days later, participants read a news report with misleading post-event information (PEI) and, after another 3 days, completed a free recall and a recognition test. The Spoken-SAI^©^ required less time to be completed than the Typed-SAI^©^ but elicited accounts with a comparable amount of correct information and accuracy. Providing an initial account using the SAI^©^ (vs. no-SAI^©^) produced more detailed accounts 1 week later regardless of response modality but did not reduce the susceptibility to misleading PEI. This provides valuable insight for improving the SAI^©^ and its applicability.

## Introduction

Interviewing witnesses can be a key procedure during police investigations, particularly by assisting in their early stages (e.g. identifying possible suspects or the location of other types of evidence). Thus, the information obtained from witnesses can determine the success of an investigation (Fisher, 2010). However, memory is fallible, and what witnesses report seldom corresponds fully with the witnessed event because witnesses can omit information and produce errors (Paulo et al., 2013, 2014). Further, the use of appropriate interviewing techniques plays a major role in obtaining accurate and informative testimony. To improve police investigations by developing science-based techniques to enhance eyewitness memory, Geiselman et al. (1984) developed the Cognitive Interview that initially included four cognitive mnemonics: report everything, mental reinstatement of context, change order and change perspective. Fisher and Geiselman (1992) later reviewed the Cognitive Interview by adding social and communicative components that are key to obtaining accurate and informative accounts and named this revised version the Enhanced Cognitive Interview (CI).

Several studies have found that the CI can increase the amount of correct information recalled by witnesses while maintaining high accuracy (Centofanti & Reece, 2006). Therefore, the CI has been widely used in police investigations (Paulo et al., 2013). However, conducting a CI shortly after the crime is not always possible due to constraints such as the lack of time and police resources (e.g. having multiple interviewers for interviewing several witnesses). In this event, police forces may have to identify the key witnesses that must be interviewed first and interview the remaining witnesses later. This can be problematic because it limits the immediate information the police is able to obtain (Hope et al., 2011). Further, this increases the likelihood of witnesses who are interviewed days or weeks after the event forgetting relevant details (Fisher, 2010; Gabbert et al., 2009) or being exposed to misleading post-event information (PEI) that may influence their accounts (Gabbert et al., 2003; Tuckey & Brewer, 2003).

However, an early opportunity to recall information about an event can help to address this issue by reducing forgetting (Brock et al., 1999) and strengthening episodic memory, thus facilitating later remembering (Shaw et al., 1995). That is, retrieving a given memory can increase its activation level and access to associated memories, thus strengthening their representation in memory and increasing the likelihood of these (and other associated) memories being recalled in a later interview (Ayers & Reder, 1998). This process occurs for both correct and incorrect information. Pickel (2004) found that if incorrect information is recalled in an initial retrieval attempt, it tends to be repeated in subsequent retrievals. Thus, an early recall opportunity is not beneficial per se. The account must be obtained using appropriate retrieval strategies that produce detailed and accurate reports. Further, detailed and accurate accounts can strengthen episodic memory access, making witnesses less susceptible to misleading PEI that they may be exposed to (Loftus, 2005).

### Self-Administered Interview (SAI^©^)

Based on these assumptions, Gabbert et al. (2009; see also Hope et al., 2011) developed the Self-Administered Interview (SAI^©^). The SAI^©^ is intended to be used when a formal interview cannot be conducted shortly after the crime. It consists of a recall tool designed to obtain comprehensive initial statements from witnesses almost immediately or shortly after the crime. It takes the form of a paper-and-pencil booklet and draws on cognitive mnemonics of the CI, such as mental reinstatement of context and the report everything mnemonic. The mental reinstatement of context consists of asking witnesses to mentally recreate the physical context of the crime as well as their physiological, cognitive and emotional states at the time of the crime. The report everything mnemonic consists of instructing witnesses to report everything they can remember with as much detail as possible, whether it seems trivial or not (Paulo et al., 2014). Moreover, the SAI^©^ is a generic recall tool in that it is suitable for obtaining information about a large variety of crimes/events while also allowing witnesses to provide, in their own words, a full account of the event without the need for an interviewer (Gabbert et al., 2012).

Several studies found that participants who completed the SAI^©^ immediately after watching a mock crime video recalled more correct information than participants who provided an immediate free recall (Gabbert et al., 2009; Gawrylowicz et al., 2014; Hope et al., 2014). The SAI^©^ allows witnesses to immediately provide time-sensitive information to the police but also enhances witnesses’ subsequent statements. Participants who completed the SAI^©^ also recalled more correct information in a subsequent retrieval attempt than participants who provided a free recall or did not provide an initial account. This was found when this second recall occurred either 1 or 2 weeks after witnessing the mock crime (Gabbert et al., 2009, 2012; Gawrylowicz et al., 2014; Hope et al., 2014; Horry et al., 2021; Paterson et al., 2015). These results support a more detailed initial account (e.g. when using the SAI^©^) having a stronger positive impact on performance in a subsequent retrieval attempt than a less detailed initial account (e.g. when using a free recall task). Allowing eyewitnesses to rehearse their memory in detail during an initial recall attempt seems to strengthen their memory and reduce forgetting (Gabbert et al., 2009). More recently, Gabbert et al. (2022) adapted the SAI^©^ to a digital format (computer and mobile application) and, across two experiments, found these digital versions to be as effective as the paper-based format in eliciting detailed initial statements and enhancing recall in a subsequent interview.

### Misleading post-event information

The SAI^©^ can also protect against exposure to misleading PEI. Gabbert et al. (2012) found that participants who completed the SAI^©^ after witnessing an event were significantly more resistant to misleading PEI introduced through a mock news report (Study 1) and misleading questions (Study 2) than participants who did not complete the SAI^©^. Nonetheless, this protective effect has been challenged in other studies. Roos af Hjelmsäter et al. (2012) and Mackay and Paterson (2015) found the SAI^©^ was not effective in reducing the susceptibility to misleading PEI introduced through co-witness discussion or an audio discussion, respectively. Mackay and Paterson (2015) argued that methodological differences may explain the different results. Roos af Hjelmsäter et al. (2012) used a modified version of the SAI^©^ containing a different structure and recall instructions, and Mackay and Paterson (2015) imposed a time limit on the completion of the SAI^©^ that may have impaired the quantity of information recalled using the SAI^©^ and its capacity to reduce the susceptibility to misleading PEI. Further, misleading PEI has been introduced differently across these studies, with the literature suggesting that the method used to introduce misleading information can affect the likelihood of that information being accepted and later recalled (Gabbert et al., 2004; Paterson & Kemp, 2006). Research has shown that misleading PEI introduced through a social source (e.g. co-witness discussion) produces a stronger misinformation effect than when PEI is introduced through a non-social source (e.g. mock news report; Gabbert et al., 2004; Paterson & Kemp, 2006). Gabbert et al. (2004) argue that social sources convey additional information, such as non-verbal cues (e.g. eye contact, facial expressions) and subtle social cues (e.g. perceived source credibility, trustworthiness) that may impact how likely witnesses are to accept this information. Therefore, it is possible that the SAI^©^ can only protect memory against misleading PEI when we have certain sources of PEI that lead to a weaker misinformation effect.

### Response modality

The use of SAI^©^ is not free from practical limitations. Despite recent studies (Gabbert et al., 2022), the SAI^©^ still requires witnesses to provide a written statement with as much detail as possible, which can take more time than witnesses are willing to dedicate to the task, possibly leading to witnesses omitting relevant information. Further, witnesses with low literacy or lack of confidence in written expression may be incapable or reluctant to complete the SAI^©^ (Hope et al., 2011). However, the effect of response modality on eyewitness memory received little attention from researchers. Bekerian and Dennett (1990) found that a spoken statement was more accurate and took significantly less time than a written statement. Likewise, Sauerland and Sporer (2011) found that a spoken statement produced more information, particularly central information about the crime and the perpetrator, than a written statement, with no differences in report accuracy. However, Lipton (1977) found no differences between these modalities regarding the accuracy and the amount of information recalled. Likewise, McPhee et al. (2014) found that both modalities produced similar accounts in an immediate recall task. Nonetheless, participants reported to prefer providing a spoken statement due to the task requiring less time and effort. More recently, Miura and Matsuo (2021) found that participants who provided a written account using the SAI^©^ recalled more correct information than participants who provided a spoken account, with similar report accuracy. However, the authors acknowledged that some methodological aspects should be considered when interpreting these results. Firstly, the way in which the SAI^©^ instructions were provided to participants was also different across conditions. While in the written modality condition, the participants received the SAI^©^ booklet and could read the instructions multiple times, in the spoken modality condition the instructions were verbally provided by the interviewer. Secondly, this study was conducted in a logographic language context, in which repeated writing is a common strategy for learning letters and characters (Naka, 1998), creating a link between writing and memorization. Thus, the advantages of the written modality might be culturally specific. Thirdly, in the sketch section of the SAI^©^ (Section C), participants in the spoken modality were only asked to picture the crime scene in their minds and verbally report that mental picture, instead of drawing it on paper as in the written modality. This might have interfered with the effectiveness of this procedure in enhancing memory recall, as supported by previous studies. Matsuo and Miura (2017) found no differences between the SAI, the CI and a free recall regarding the amount of correct information recalled when the sketch section of the SAI was excluded from the analysis. However, when the information provided in this section was included, the amount of correct information recalled in the SAI was greater than in the CI and the free recall. Likewise, Maras et al. (2014) found that the sketch section of the SAI^©^ elicited more correct information than a corresponding section where participants were asked to provided details of the scene without the possibility of producing a sketch. Thus, the benefit of providing a written account when using the SAI^©^ found by Miura and Matsuo (2021) might have been affected by these other factors. In sum, mixed results make it difficult to determine what response modality is more effective in improving memory recall when using the SAI^©^.

Nevertheless, there are distinct reasons that support collecting a spoken account instead of a written statement. For instance, difficulties that certain witnesses might experience while writing (e.g. spelling and grammar) can be mitigated by requesting a spoken statement instead (McPhee et al., 2014). Further, the effortful nature of writing in comparison with speaking may influence the amount of information a witness is willing to report. Speaking also requires less time and effort than writing because writing is typically less practised and may not be as automatized. Thus, writing demands more working memory resources than speaking (Bourdin & Fayol, 1994; Kellogg, 2007). It is also important to consider that handwriting, which is used in the SAI^©^, is now less common due to the increasing use of digital communication, with several studies finding typing to be significantly faster than handwriting (Bouriga & Olive, 2021; Bui et al., 2013). Although it is plausible to assume that some of the limitations mentioned above regarding handwriting (e.g. difficulties with spelling and grammar; requiring more effort than speaking) are also applicable to typing, previous research has not examined the effectiveness of a spoken modality in comparison with a typed response to the SAI^©^. Therefore, our study addressed this by testing whether response modality to the SAI^©^ (spoken vs. typed) affects how accurate and detailed initial accounts are and whether this has an impact on the protective effect the SAI^©^ has on a subsequent free recall task.

### Current study

In the present study, we tested the effect of response modality (spoken vs. typed) used to complete the SAI^©^ on the amount of correct information recalled, report accuracy and the time required to provide an initial statement. Further, we tested whether completing the SAI^©^ affected a subsequent free recall task (i.e. reduce forgetting and reduce the acceptance of misleading PEI) and whether this protective effect interacts with response modality. Four main hypotheses were established: (a) participants who provided a spoken account using the SAI^©^ would recall more correct information (Kellogg, 2007; Sauerland & Sporer, 2011) and faster (McPhee et al., 2014) than participants who provided a typed account; (b) participants who provided an initial account using the SAI^©^ (spoken or typed) would recall more correct information 1 week later in a second recall task than the control group who did not provide an initial account (Gabbert et al., 2009; Hope et al., 2014); (c) participants who provided an initial account using the SAI^©^ would also recall/select less misleading PEI in the subsequent retrieval tasks (free recall and multiple-choice recognition test, respectively; Gabbert et al., 2012); and (d) considering the protective effect an initial retrieval attempt has in a subsequent retrieval attempt is linked to the quantity of correct information initially recalled (Gabbert et al., 2009), we expected this protective effect (operationalized in Hypotheses b and c) to be stronger for participants who provide a spoken account.

## Method

### Participants

An *a priori* power analysis was conducted using G*Power 3.1 (Faul et al., 2009) to calculate the minimum sample size necessary to test the difference between the three groups using analysis of variance. For this analysis, we used an alpha of .05 and the smallest effect size (Cohen’s *d*** **=** **0.66) reported in the literature for the difference in correct recall between participants who completed the SAI^©^ and participants who did not (control group) in a subsequent retrieval attempt (Hope et al., 2014). Results showed that 31 participants per group would be required to achieve a power of .80. A total of 96 Portuguese university students participated in this study for course credits. Three participants were excluded because they did not return for the third session. Thus, 93 participants, 80 female and 13 male, aged 18–33 years (*M*** **=** **20.38, *SD*** **=** **3.19) were included in the analysis. Seventy-one were psychology students, and 22 were criminology students. Participants were randomly assigned to one of the three interview conditions with 31 participants each. The Typed-SAI^©^ condition had 27 female participants and four male participants aged 18–33 years (*M*** **=** **20.39, *SD*** **=** **3.39). The Spoken-SAI^©^ condition had 27 female participants and four male participants aged 18–30 years (*M*** **=** **20.19, *SD*** **=** **2.60). The no-SAI^©^ condition had 26 female participants and five male participants aged 18–32 years (*M*** **=** **20.55, *SD*** **=** **3.58). Participants were recruited via email or through the course credit system implemented in their university.

### Design

A between-subjects design was used with SAI^©^’s response modality as the independent variable with three conditions: Typed-SAI^©^; Spoken-SAI^©^; and no-SAI^©^ (control group). In the first retrieval attempt, the dependent variables were: (a) the amount of correct information recalled – that is, the number of correct units of information recalled; (b) report accuracy – that is, the ratio between the number of correct units of information recalled over the total number of units of information recalled; and (c) the time taken by each participant to complete the SAI^©^, measured in minutes. In the second retrieval attempt, the dependent variables were: (a) the amount of correct information recalled; (b) report accuracy; and (c) the number of units of misleading PEI recalled, measured in units of information. Finally, in the recognition test, the dependent variable was accuracy – that is, the ratio between the number of correct answers over the total number of answers.

### Materials

#### Mock crime video

A non-violent video edited from the Portuguese film ‘Sorte Nula’ was used (Fragata, 2004). This video was 2 min and 58 s long and contained varied and substantial information regarding the different forensically relevant categories of information described in the coding section. This non-violent video recording shows the concealment and transport of a corpse by two men, both physically and verbally interacting.

#### Picture Source Monitoring task

All participants completed a Picture Source Monitoring task as a distraction task after watching the video. This was based on the task used by Krix et al. (2015), and all pictures were taken from Snodgrass and Vanderwart (1980). Firstly, participants were shown 28 pictures one at a time. Each picture was displayed for 3 s in one of the four quadrants of the screen. Participants were asked to pay attention to both the pictures presented and the quadrant in which they appeared. Then, these 28 pictures (targets) and 28 new pictures (distractors) were presented in a counterbalanced way in the centre of the screen. For each picture, participants had to decide whether it was a previously presented picture (target) or not (distractor). If they answered that the picture was a target, the participant was also asked to identify the quadrant in which the picture was presented earlier.

#### Self-Administered Interview (SAI^©^)

The SAI^©^ (Gabbert et al., 2009) is a recall tool that comprises several sections containing instructions designed to facilitate the recall of a witnessed event. Section A instructs the witness to provide a detailed account without guessing and contains information and instructions pertaining to the use of the Mental Reinstatement of Context and Report Everything mnemonics. Section B asks for detailed information about the perpetrator’s appearance (e.g. hair, height, clothing). Section C asks the witness to sketch the crime scene to elicit relevant spatial information. Section D encourages witnesses to describe any persons who may have been present and may have seen what happened. Section E requests detailed information about any vehicles that may have been involved in the incident (e.g. size, shape, colour, etc.). Section F prompts witnesses to recall additional information that may not have been reported in the previous sections. For a full description of the SAI^©^, one can read Gabbert et al. (2009) and Hope et al. (2011).

The original version of the SAI^©^ was translated to European Portuguese and adapted to a digital platform that allowed online data collection: Jotform (https://eu.jotform.com/). The layout of the SAI^©^ and the number/order of pages were analogous to those in the original version, with the only difference being that the online version allowed participants to access the SAI^©^ using their computers and type their responses using their keyboards. This platform also measured the time taken by each participant to complete the SAI^©^. Participants in the Spoken-SAI^©^ condition received the same online version of the SAI^©^ but were asked to provide a spoken response instead that was audio-recorded using the Zoom Video Conferencing platform (Version 5.6.1). For Section C of the SAI^©^ (sketch), participants used a digital drawing tool included in the booklet and mimicking the original layout of the SAI^©^. Participants could draw a sketch using the keyboard and mouse, and, as in the original version of the SAI^©^, participants could also provide labels and notes to indicate features of the scene or any aspects of the sketch that they were uncertain about. Participants in the Spoken-SAI^©^ condition were asked to verbalize these labels and notes instead of typing them (Typed-SAI^©^ condition). All other instructions were the same for both conditions. This ensured the SAI^©^ protocols were as close as possible to the original paper booklet, but also consistent with participants’ response modality.

#### Post-event information (PEI)

The PEI was introduced through a mock news report, which briefly summarized the incident depicted on the video. We mimicked the logo and layout of a credible national journal. The news report included eight units of misleading PEI (see Table 1) and correct PEI (i.e. information consistent with what participants watched in the video) necessary to write a realistic and intelligible news report. This source of PEI and other procedural decisions (e.g. the retention intervals and the moment the PEI was introduced) were tested in a pilot study reported in the Supplementary Materials.

**Table 1.** Misleading PEI items presented in the news report.

Item	Video	News report
Corpse	Man	Woman
Perpetrator’s hair colour	Brown	Blond
Weapon’s colour	Black	Silver
Bag	Plastic	Cloth
Car trunk	Open	Closed
Corpse transported by	Two perpetrators	One of the perpetrators
Perpetrators agree to meet at	School	Coffee shop
Tool for burying the body	Shovel	Hoe

Note: PEI = post-event information.

#### Free recall task

All participants completed a spoken free recall where an interviewer asked them to report everything they could remember about the event in any chronological order and at their desired pace. The following initial instructions and procedures considered best practice for interviewing cooperative witnesses were given to all participants (Fisher & Geiselman, 1992; Paulo et al., 2016). The interviewer explained the purpose of the interview (i.e. gather as much information as possible about the event depicted in the video) and asked participants to provide as much detail as possible, even partial or minor details (report everything instruction). Participants were asked not to guess. Further, participants were informed they could take as much time as needed and that they could start, pause and stop their report at any time (transfer of control to the eyewitness). Lastly, mental reinstatement of context was applied. Written instructions were followed and read verbatim. All instructions for the free recall task are available in the Supplementary Materials.

#### Recognition test

Participants completed a multiple-choice recognition test with 24 multiple-choice questions, each containing two response options (one of which was the correct option for all participants). Eight questions addressed information that was only presented in the mock crime video for all participants (e.g. ‘The corpse had a ____ sweater: (a) White; (b) Black’). Eight questions addressed information presented in the video that was consistent with the news report for all participants (e.g. ‘The weapon was a ____: (a) Shotgun; (b) Pistol’). Eight questions addressed information presented only in the video for the participants who did not receive misleading PEI but containing the incorrect response option that matched the misleading PEI presented in the news report for the misleading PEI groups (e.g. ‘The corpse had a bag made of ____: (a) Cloth; (b) Plastic’).

### Procedure

Data collection was conducted using Zoom Video Conferencing platform (Version 5.6.1). Ethics committee approval was obtained. Having read information about the study and having signed a consent form, participants individually took part in three sessions.

#### Session 1

Participants were shown the video recording after being randomly assigned to one of the three interview conditions (Typed-SAI^©^ vs. Spoken-SAI^©^ vs. no-SAI^©^). Using the screen share functionality, participants were asked to pay as much attention as possible to the video recording because they would later be asked to recall the event. All participants confirmed they had not seen the video recording before. A 30-min retention interval was then introduced (Sauerland & Sporer, 2011) to mimic the approximate time the police would need to arrive at the crime scene and distribute the SAI^©^. During this retention interval, participants were involved in three tasks: (a) they completed a socio-demographic questionnaire (e.g. age, gender, course); (b) they completed a Picture Source Monitoring task (Krix et al., 2015; Snodgrass & Vanderwart, 1980); and (c) they had an informal conversation with the researcher regarding neutral topics until the 30-min retention interval was completed.

Following this, participants provided a typed account using the SAI^©^ (Typed-SAI^©^ condition), a spoken account using the SAI^©^ (Spoken-SAI^©^ condition) or no account at all (no-SAI^©^ condition: control group). Participants who completed the SAI^©^ were informed they would have to carefully follow the instructions provided in the online booklet, and no time restrictions were imposed. Given the self-administered format of the SAI^©^, participants in both response conditions (Spoken and Typed) switched off their cameras while completing the SAI^©^ to allow for full concentration and a more relaxed environment. For similar reasons, the researcher also kept the camera and microphone turned off for both conditions but informed participants he would be online and available to respond to any possible queries concerning the completion of the SAI^©^.

#### Session 2

The second session took place 4 days after Session 1. All participants were informed they would read a news report about the crime they witnessed 4 days before. This news report contained misleading PEI and was shown using the screen share functionality. The researcher was present in the call and asked participants to read the news report aloud to ensure participants were engaged in the task.

#### Session 3

The third session took place 3 days after the second session (1 week after seeing the video). The interviewer requested participants to provide a spoken free recall. Participants were asked to recall what they could remember about the video they had watched in Session 1, using the recall instructions described in the Materials section. This session was audio recorded to allow data scoring and analysis. After completing the free recall task, participants completed the recognition test using Google Forms. The researcher sent the recognition test link through the Zoom Chat. Participants were asked to answer this test according to what they saw in the video. Finally, participants were then thanked for their participation and debriefed.

### Coding

All authors first compiled and agreed upon a comprehensive list of details in the video recording. Three hundred and twenty-three relevant units of information were identified and categorized as referring to (a) a person; (b) an action; (c) an object; (d) a location; and (e) a conversation or sound. Concerning the recall tasks in both retrieval attempts (initial recall using the SAI^©^ and subsequent free recall), the units of information were classified as correct (e.g. saying that the sweater was white when it was white), incorrect (e.g. saying that the sweater was white when it was black) or confabulation (e.g. mentioning that there was a bus when it did not exist). This list also included the correct and misleading PEI items presented to participants in the news report. Units of information referring to the misleading PEI were classified as either incorrect (i.e. when participants recalled the item of misleading PEI) or correct (i.e. if participants resisted recalling the item of misleading PEI and instead recalled the correct unit of information presented in the video recording). Each unit of information was only coded the first time it was mentioned, and subjective statements or opinions were not coded (e.g. ‘he was gorgeous’).

### Inter-rater reliability

To assess the inter-rater reliability when coding the recall tasks in both retrieval attempts (initial recall using the SAI^©^ and subsequent free recall), 30 (19.35%) randomly selected interviews (15 from each researcher and six from each interview condition/retrieval attempt) were coded independently by two researchers. Intraclass correlation coefficients (ICCs) were calculated for the total of information recalled, correct information recalled (for both retrieval attempts) and misleading PEI recalled (for the second recall only). High inter-rater reliability was found for all measures in that ICC values ranged between .998 and 1.000.

## Results

### First retrieval attempt (SAI^©^)

Independent *t* tests were conducted to compare the number of correct units of information recalled, accuracy (i.e. the ratio between the number of correct units of information recalled over the total number of units of information recalled) and the time taken to complete the SAI^©^ across the two groups who provided this initial recall attempt (Spoken-SAI^©^ and Typed-SAI^©^). Table 2 shows the descriptive statistics for these measures.

**Table 2.** Number of correct units of information recalled, accuracy and time taken to complete the SAI^©^ according to response modality.

Response modality	Correct information			Accuracy			Time		
	*M*	*SD*	95% CI	*M*	*SD*	95% CI	*M*	*SD*	95% CI
Spoken-SAI^©^	80.87	24.15	[72.01, 89.73]	.91	.03	[.90, .92]	18.14	5.82	[16.00, 20.27]
Typed-SAI^©^	73.26	16.05	[67.37, 79.14]	.90	.04	[.89, .91]	30.49	11.22	[26.37, 34.60]
Total	77.06	20.69	[71.81, 82,32]	.90	.03	[.90, .91]	24.31	10.83	[21.56, 27.06]

Note. SAI^©^ = Self-Administered Interview; CI = confidence interval.

#### Correct information

There was no significant difference between the Spoken-SAI^©^ and Typed-SAI^©^ conditions regarding the number of correct units of information recalled, *t*(60) = 1.46, *p* = .149, Cohen’s *d*** **=** **0.37 (95% confidence interval, CI [−0.13, 0.87]).

#### Accuracy

There was no significant difference between the Spoken-SAI^©^ and the Typed-SAI^©^ conditions regarding report accuracy, *t*(60) = 1.08, *p* = .286, Cohen’s *d*** **=** **0.27 (95% CI [−0.23, 0.77]).

#### Time taken to complete the SAI^©^

Participants in the Spoken-SAI^©^ condition took significantly less time (measured in minutes) to complete the SAI^©^ than those in the Typed-SAI^©^ condition, *t*(60) = 5.44, *p* < .001, Cohen’s *d*** **=** **1.38 (95% CI [0.82, 1.93]). As shown in Table 2, participants in the Spoken-SAI^©^ condition required, on average, approximately 12 min less to complete the SAI^©^ than the participants in the Typed-SAI^©^ condition.

### Second retrieval attempt (free recall)

Three independent one-way analyses of variance (ANOVAs) were conducted to see if response modality in the first retrieval attempt (Spoken-SAI^©^, Typed-SAI^©^, No-SAI^©^) had an effect on the number of correct units of information recalled, accuracy and the number of units of misleading PEI recalled in the free recall task (see Table 3 for descriptive statistics).

**Table 3.** Number of correct units of information recalled, accuracy and misleading PEI recalled according to response modality.

Response modality	Correct information			Accuracy			Misleading PEI		
	*M*	*SD*	95% CI	*M*	*SD*	95% CI	*M*	*SD*	95% CI
No-SAI^©^ (control group)	50.97	17.63	[44.50, 57.44]	.87	.07	[.84, .89]	0.90	1.08	[0.51, 1.30]
Spoken-SAI^©^	70.16	21.48	[62.28, 78.04]	.90	.05	[.88, .92]	0.55	0.85	[0.24, 0.86]
Typed-SAI^©^	67.10	17.69	[60.61, 73.58]	.88	.05	[.86, .90]	0.58	0.72	[0.32, 0.84]
Total	62.74	20.63	[58.49, 66,99]	.88	.06	[.87, .89]	0.68	0.90	[0.49, 0.86]

Note. PEI = post-event information; SAI^©^ = Self-Administered Interview; CI = confidence interval.

The number of misleading PEI can range from 0 to 8.

#### Correct information

A significant effect of the SAI’s response modality on the number of correct units of information recalled during the (delayed) free recall task was found, *F*(2, 90) = 9.11, *p* < .001, $\eta_{p}^{2}$ = .17. Participants in the No-SAI^©^ condition recalled significantly fewer correct units of information than participants in the Spoken-SAI^©^ condition, *t*(60) = 3.84, *p* < .001, Cohen’s *d*** **=** **0.98 (95% CI [0.45, 1.50]) and participants in the Typed-SAI^©^ condition, *t*(60) = 3.60, *p* < .001, Cohen’s *d*** **=** **0.91 (95% CI [0.39, 1.43]). However, there were no differences between participants in the Spoken-SAI^©^ and Typed-SAI^©^ conditions, *t*(60) = 0.61, *p*** **=** **.542, Cohen’s *d*** **=** **0.16 (95% CI [−0.34, 0.65]).

#### Accuracy

No effect of the SAI’s response modality on the accuracy in the delayed free recall task was found, *F*(2, 90) = 2.33, *p* = .103, $\eta_{p}^{2}$ = .05.

#### Misleading PEI recalled

No effect of the SAI’s response modality on the number of units of misleading PEI recalled during the free recall task was found, *F*(2, 90) = 1.49, *p* = .230, $\eta_{p}^{2}$ = .03. As shown in Table 3, the number of misleading PEI recalled in the free recall task was very low (less than one unit of information) for all three groups.

#### New versus repeated information

To determine the extent to which completing the SAI^©^ (Spoken and Typed) in the first retrieval attempt reduces forgetting and preserves the information initially recalled, we scored the units of information recalled during the second retrieval attempt (correct and incorrect) as either ‘new’ or ‘repeated’. New units of information had not been recalled in the first retrieval attempt using the SAI^©^. Repeated information had already been recalled in the first retrieval attempt using the SAI^©^ and could follow four patterns: (a) correct–correct, if a unit of information (e.g. colour of the car) was recalled correctly in both attempts; (b) incorrect–correct, if recalled incorrectly in the SAI^©^ but rectified in the free recall; (c) correct–incorrect, if initially recalled correctly in the SAI^©^ but incorrectly in the free recall; and (d) incorrect–incorrect, if recalled incorrectly in both attempts.

Regardless of response modality, 67% of the correct units of information recalled in the first retrieval attempt using the SAI^©^ were also recalled in the second retrieval attempt. Further, 55% of the incorrect units of information recalled in the first retrieval attempt using the SAI^©^ were also recalled in the second retrieval. New units of information represented a smaller percentage of the total units of information recalled in the second retrieval attempt, both for correct recall (21%) and for incorrect recall (5%). Only a fractional number of units of information were correctly recalled in the first retrieval attempt and then incorrectly reported/altered in a second retrieval attempt or vice-versa (see Table 4). No differences between the Spoken SAI^©^ and Typed SAI^©^ conditions were found for any of the measures reported in Table 4 (all *p* values > .05).

**Table 4.** Number of new and repeated units of information recalled in the free recall task according to response modality.

	Repeated information								New information			
	Correct– correct		Incorrect– correct		Correct– incorrect		Incorrect– incorrect		Correct		Incorrect	
	*M*	*SD*	*M*	*SD*	*M*	*SD*	*M*	*SD*	*M*	*SD*	*M*	*SD*
Spoken-SAI^©^	54.26	19.52	0.42	0.85	0.81	0.98	4.07	2.22	15.45	6.05	2.65	1.70
Typed-SAI^©^	49.87	14.49	0.39	0.72	0.58	0.67	4.36	2.76	16.77	8.05	4.26	3.01
Total	52.07	17.19	0.40	0.78	0.69	0.84	4.21	2.49	16.11	7.10	3.45	2.56

Note. SAI^©^ = Self-Administered Interview.

### Third retrieval attempt (recognition test)

We conducted one 3 × 3 ANOVA to explore the effects of SAI’s response modality (Spoken vs. Typed vs. control group) and type of information addressed in the questions (information that was only presented in the video versus information presented in the video that was consistent with the news report versus questions containing an incorrect response option that matched misleading PEI) on participants’ accuracy in the recognition test. Accuracy was measured by calculating the ratio between the number of correct answers over the total number of answers.

We found a significant main effect of SAI’s response modality on participants’ accuracy in the recognition test, *F*(2, 90) = 6.51, *p* = .002, $\eta_{p}^{2}$ = .13. Participants in the control group (*M* = .74, *SD* = .09, 95% CI [.70, .77]) were less accurate than those in the Typed-SAI^©^ (*M* = .82, *SD* = .10, 95% CI [.78, .85]), *t*(60) = 3.30, *p* = .004, Cohen’s *d*** **=** **0.34, and Spoken-SAI^©^ conditions (*M* = .81, *SD* = .09, 95% CI [.77, .84]), *t*(60) = 2.91, *p* = .014, Cohen’s *d*** **=** **0.30. However, no significant difference was found between the Spoken-SAI^©^ and Typed-SAI^©^ conditions, *t*(60) = 0.40, *p*** **=** **1.000, Cohen’s *d*** **=** **0.04.

We also found a significant main effect of the type of information addressed in the questions on participants’ accuracy, *F*(1.876, 168.797) = 34.42, *p* < .001, $\eta_{p}^{2}$ = .28. The accuracy in the questions concerning items presented in the video that were consistent with the news report (*M* = .87, *SD* = .12, 95% CI [.84, .89]) was higher than in the questions about items only presented in the video (*M* = .80, *SD* = .14, 95% CI [.77, .83]), *t*(92) = 3.06, *p* = .008, Cohen’s *d*** **=** **0.32, and questions about the items of misleading PEI (*M* = .69, *SD* = .21, 95% CI [.64, .73]), *t*(92) = 8.21, *p* < .001, Cohen’s *d*** **=** **0.85. The accuracy in the questions about items only presented in the video was also higher than that in the questions about the items of misleading PEI, *t*(92) = 5.15, *p* < .001, Cohen’s *d*** **=** **0.53.

There was no interaction effect between SAI’S response modality and the type of information addressed in the questions, *F*(3.751, 168.797) = 1.22, *p* = .303, $\eta_{p}^{2}$ = .03.

## Discussion

This study tested the effect of the response modality used to complete the SAI^©^ on the amount of correct information recalled, report accuracy and the time required to provide an initial statement. Further, this study tested whether completing the SAI^©^ affected a subsequent free recall and recognition task (i.e. reduce forgetting and reduce the acceptance of misleading PEI) and whether this protective effect interacts with response modality.

Contrary to our initial hypothesis, there was no significant effect of response modality on the amount of correct information recalled in the first retrieval attempt. However, as expected, participants in the Spoken-SAI^©^ condition took significantly less time to complete the SAI^©^ than those in the Typed-SAI^©^ condition (approximately 12 min less). Therefore, a spoken recall can have advantages in terms of requiring less time and allowing reports to be collected promptly. Report accuracy was high and similar for both conditions, similarly to McPhee et al. (2014), who found no differences between the spoken and handwritten modalities concerning the accuracy and the amount of correct information recalled in an immediate recall task. Although we expected the spoken modality to produce more detailed accounts (Kellogg, 2007; Sauerland & Sporer, 2011), there are plausible explanations for why our results did not support our initial hypothesis. The advantages of a spoken account may become more prominent when the event to be recalled is more extensive or more detailed, therefore requiring more time to be recalled. This is supported by Sauerland and Sporer (2011) who used a 6:30-min video and found that a spoken statement produced more information than a written statement. In contrast, we used a short mock crime video (approximately 3 min) and did not find the spoken modality to elicit more information than a typed modality. It is possible that when a more complex/detailed event is shown to participants, the laborious task of handwriting or typing a longer account becomes more detrimental. This might therefore interfere with the witness’s motivation to dedicate the time and effort necessary to handwrite/type all the relevant information with as much detail as possible in comparison with a spoken account that requires less time and effort. This is compatible with the results found by Gabbert et al. (2022) who also used a short video (2:12 min) and found no advantage of a typed account in terms of eliciting more information from witnesses in comparison with a handwritten account that should require more time and effort.

Another possible explanation for both response modalities producing reports with a similar level of detail could concern the writing method (i.e. typing) and the characteristics of the sample used in this study. In the studies that found differences concerning the level of detail elicited between the spoken and written modalities, the written modality was operationalized through handwriting (Bekerian & Dennett, 1990; Sauerland & Sporer, 2011). However, typing is significantly faster than handwriting (Bouriga & Olive, 2021; Bui et al., 2013). Further, in the educational and academic context, it is increasingly common for students, especially university students, to use computers to take notes during classes, and according to Bouriga and Olive (2021), it is now more frequent than handwriting. Given that the sample of the present study consisted of university students, typically proficient in typing, it is possible that typing did not require as much of an additional effort (e.g. physical and cognitive) as other methods of writing (e.g. handwriting) would, resulting in no differences between both modalities (spoken and typed) concerning the amount of correct information recalled. Future studies could explore the differences between these two modalities with participants who are less trained in typing and/or using digital skills, possibly also comparing these digital versions with the original version of the SAI^©^ where the instructions are provided in the form of a paper-and-pencil booklet instead of a digital form.

As expected, participants who provided an initial account using the SAI^©^ recalled more correct information in a second recall 1 week later than participants who did not have an initial recall opportunity (control group). This is consistent with previous research showing that an early opportunity to provide a detailed initial account (e.g. using the SAI^©^) increases the quantity of correct information recalled in a subsequent retrieval attempt in comparison with a less detailed initial account (e.g. obtained using a free recall task) or no initial account (Gabbert et al., 2009; Hope et al., 2014). Contrary to our initial hypothesis, this protective effect was not influenced by response modality – that is, the spoken modality did not further increase recall during a second recall attempt. This can be due to both modalities unexpectedly producing accounts with comparable levels of detail during the first retrieval attempt (discussed above). Considering that the positive impact that an initial recall opportunity has on a subsequent recall is related to the amount of information recalled in the initial recall (Hope et al., 2014), it is expected that accounts with a similar level of detail will produce a similar impact on a subsequent recall, therefore explaining our results.

To further explore the impact of an initial retrieval attempt using the SAI^©^ on a subsequent retrieval attempt, we examined the consistency between the information recalled in both attempts. Regardless of response modality, most of the correct information (67%) recalled in the first retrieval attempt was also recalled in the second retrieval attempt, suggesting that a substantial number of correct units of information were preserved over the 1-week retention interval. However, this also occurred for incorrect information, with 55% of the incorrect units of information recalled in the first retrieval attempt being recalled 1 week later. This supports previous research suggesting that an early recall opportunity can reduce forgetting (Brock et al., 1999) and increase the likelihood of these initially recalled memories being recalled in a later interview. However, this process also occurs for errors (Pickel, 2004). Therefore, an initial recall is not beneficial per se – that is, it must be obtained using appropriate retrieval strategies that produce detailed and accurate reports. Further, 21% of the units of information recalled in the second retrieval attempt were new correct units of information, suggesting that a formal police interview is important to obtain new information that was not captured in the initial self-report. This supports that the SAI^©^ should not replace a subsequent formal interview, as stated by its authors (Gabbert et al., 2009). Nevertheless, the introduction of PEI (correct and misleading) should be taken into consideration when interpreting these results because the protective effect of an initial retrieval attempt using the SAI^©^ and the effects of providing PEI might have been confounded.

This study also investigated whether completing the SAI^©^ protects against exposure to misleading PEI. As in Roos af Hjelmsäter et al. (2012) and Mackay and Paterson (2015), we found participants who completed the SAI^©^ were no less susceptible to misleading PEI than participants who did not complete the SAI^©^, regardless of response modality. These results differ from other studies where conducting the SAI^©^ decreased susceptibility to misleading PEI (Gabbert et al., 2012; Gittins et al., 2015). Methodological differences between the studies may explain the different results. In the studies that found the SAI^©^ to have a protective effect against exposure to misleading PEI, misleading PEI was introduced immediately or minutes before the second retrieval (Gabbert et al., 2012; Gittins et al., 2015; Paterson et al., 2015). The present investigation introduced a retention interval of approximately 72 hours between exposure to misleading PEI and the second retrieval attempt. This procedural decision was made to increase ecological validity because it would be unlikely that in a real-life scenario a witness would be exposed to the type of misleading PEI we used (i.e. news report) immediately before being interviewed. Further, a longer retention interval between encoding the PEI and the second retrieval attempt allowed the reconsolidation process to occur. In a study by Capelo et al. (2019) on episodic memory reconsolidation, an interval between sessions of 48 h was used to guarantee the reconsolidation process to occur. However, this 72 hours retention interval may have led to the forgetting of the misleading PEI, with previous studies suggesting that PEI is more likely to be retrieved when introduced immediately (or shortly before) a retrieval task (Paterson et al., 2015). This might explain why the number of misleading PEI recalled in our study was low for all three groups (less than one unit of information in the recall task). Similarly, in the recognition test, the accuracy in the eight questions concerning the incorrect PEI was relatively high for all groups. This is compatible with the Wang et al. (2014) study, where no difference was found concerning the susceptibility to misleading PEI between participants who performed an initial recall and participants who did not. The authors used a 1 week retention interval and argued this may have led to the forgetfulness of misleading PEI, resulting in the floor effect that might have also occurred in our study. Therefore, future studies should consider how different retention intervals (between introducing the PEI and retrieving the event) can influence the protective effect of the SAI^©^.

### Conclusion and practical implications

To our knowledge, this study was the first to test whether providing a spoken response to a digital version of the SAI^©^ results in a detailed and accurate initial recall and improves recall in a subsequent interview. We found the spoken reports to be as detailed and accurate as typed accounts but considerably less time demanding. Although the SAI^©^ currently consists of a paper-and-pencil booklet that requires a written response, new technologies are increasingly an integral part of our routine. They can be used to improve these tools, namely (but not only) by allowing spoken accounts to be recorded, which can be particularly valuable for witnesses and victims who might not be able to provide written accounts (e.g. with visual impairments or low literacy). In combination with recent studies (Gabbert et al., 2022) supporting the use of the SAI^©^ in digital formats (computer or mobile applications), our study provides valuable information for developing new methods to collect initial accounts. New versions of the SAI^©^ could allow for a spoken or typed response option, allowing witnesses to choose their preferred method depending on individual preferences and needs. Ultimately, this can help promote equity when accessing police services, which is a critical concern in modern policing.

## Supplementary Material

Supplemental Material

Supplemental Material

## Data Availability

The data that support the findings of this study are openly available in Open Science Framework (OSF) at https://osf.io/c4k38/?view_only=2fbe942049a64071a16fa12bca0ff89c.
