# Supplementary material for: The effect of response modality on witness statements when using the self-administered interview
Source: Psychiatr Psychol Law. 2024 Apr 7;32(3):337–53. doi: 10.1080/13218719.2024.2313977 (PMC12123897; doi:10.1080/13218719.2024.2313977)
Supplement: Supplemental Material [file TPPL_A_2313977_SM3019.docx]

**Supplementary Materials**

Pilot Study

This pilot study aimed to inform our main experiment, namely by ensuring our procedural decisions (e.g., retention interval and the moment the PEI was introduced to participants) and materials (e.g., sources of misleading PEI) could elicit a misinformation effect. To this end, we tested the effect of two sources of misleading PEI on memory retrieval in a free recall and a multiple-choice recognition task.

**Method**

***Participants***

A total of 125 university students participated in this experiment for course credits. Five participants were excluded because they did not attend the third session. Thus, 120 students, 94 female and 26 male, aged 18 to 44 years (*M* = 20.76, *SD* = 3.74) were included in the analysis. Participants were randomly assigned to one of four groups: (1) high credibility source with misleading PEI: 25 female participants and five male participants; (2) high credibility source without misleading PEI: 22 female and eight male participants; (3) low credibility source with misleading PEI: 25 female participants and five male participants; and (4) low credibility source without misleading PEI: 22 female and eight male participants.

***Design***

A 2 (source credibility: high, low) $\times$ 2 (misleading PEI: present, absent) between-subjects design was used. The dependent variables were (1) the number of units of misleading PEI recalled in the free recall task, measured in units of information, and (2) accuracy in the recognition test. Accuracy in the recognition test was measured by calculating the ratio of correct answers over the total number of answers.

***Materials***

*Mock Crime Video*

This was the same mock crime video used in our main study (described in the article).

*Free Recall Task and Recognition Test*

All participants completed the free recall and recognition tasks following the same instructions described in the article.

*Post-event Information (PEI)*

The PEI was introduced through a mock news report which briefly summarised the incident depicted on the video. Source credibility was manipulated by changing the publisher and layout of this mock news report. We mimicked the logo and layout of a credible journal for the high credibility source and the logo and layout of an unreliable social media page and platform for the low credibility source. Each of these two sources contained one version containing eight units of misleading PEI and another without these eight units of misleading PEI. All versions of the news report included the same information and format (e.g., font size, spacing, etc.) and correct PEI (i.e., information consistent with what participants watched in the video) necessary to write a realistic and intelligible news report. However, the same eight items of misleading PEI (see Table 1) were added to the news report given to the two groups of participants in the misleading PEI present conditions (for both credibility source conditions).

[Table 1 here]

***Procedure***

Due to Covid-19 restrictions, virtual data collection was used. All sessions (see below) were completed using the Zoom Video Conferencing platform (Version 5.6.1). Ethics committee approval was obtained. Having read information about the study and signed a consent form, participants individually took part in three Zoom sessions.

*Session 1*

Participants were shown the video recording in the first session using their personal computers. Participants were asked to pay as much attention as possible to this video and were informed they would later be asked to recall what they remembered about the video event. The researcher showed the video to participants using the screen share option to ensure participants were engaged in the task and saw the video without interruptions and in an appropriate environment (e.g., a quiet space without distractions or interruptions). All participants confirmed that they had never seen this video before.

*Session 2*

The second session took place four days later. After being randomly assigned to one of the four experimental conditions (high credibility source with misleading PEI vs high credibility source without misleading PEI vs low credibility source with misleading PEI vs low credibility source without misleading PEI), participants were given the corresponding news report using the screen share option. All participants were informed they would be given a news report about the crime they witnessed four days before. The researcher was also present in the call and asked participants to read the news report aloud to ensure participants were engaged in the task.

*Session 3*

The third session took place two days after the second session. All participants completed a spoken free recall where they were asked to recall what they could remember about the video they had watched in Session 1, using the recall instructions described in the materials section. This session was audio recorded to allow data scoring and analysis. After completing the free recall task, participants completed the recognition test using Google Forms. The researcher sent the recognition test link through the Zoom Chat. Participants were asked to answer this test according to what they saw in the video. Finally, participants were thanked for their participation.

***Coding***

Interview recordings were coded using the same comprehensive list of details used in the article.

**Results**

***Free Recall***

A 2 $\times$ 2 ANOVA was conducted to test if source credibility (high vs low) and misleading PEI (present vs absent) affected the number of units of misleading PEI recalled in the free recall task. We found a significant main effect of misleading PEI on the number of units of misleading PEI recalled, *F*(1, 116) = 11.72, *p* < .001, η^2^_p_ = 0.09. Participants who read the news report containing misleading PEI recalled more units of misleading PEI than participants who saw the news report that did not contain misleading PEI (see Table 2). There was no main effect of source credibility on the number of units of misleading PEI recalled, *F*(1, 116) = 0.01, *p* = .922, η^2^_p_ = 0.00. There was no interaction between the misleading PEI and source credibility on the number of units of misleading PEI recalled in the free recall task, *F*(1, 116) = 0.09, *p* = .770, η^2^_p_ = 0.001. To select a source of misleading PEI for our main study, we compared the difference between the misleading PEI absent and misleading PEI present groups concerning the number of units of misleading PEI recalled (i.e., the misinformation effect size) for the high credibility source group, *t*(58) = 2.68, *p* = .010, Cohen’s *d =* 0.69, and for the low credibility source group, *t*(58) = 2.29, *p* = .026, Cohen’s *d* = 0.59.

[Table 2 here]

***Recognition Test***

A mixed 2 $\times$ 2 $\times$ 3 ANOVA was conducted to test the effect of source credibility (high vs low), misleading PEI (present vs absent) and type of information addressed in the questions (information that was only presented in the video vs information presented in the video that was consistent with the news report vs questions containing an incorrect response option that matched the misleading PEI) on participants’ accuracy. Accuracy was calculated by the ratio of correct answers over the total number of answers.

We found a significant main effect of misleading PEI on participants’ accuracy, *F*(1, 116) = 10.67, *p* = .001, η^2^_p_ = 0.08. Participants who saw the news report with misleading PEI were less accurate than those who saw the news report that did not contain misleading PEI (see Table 3).

The main effect of the type of information addressed in the questions was also significant, *F*(1.866, 216.469) = 14.60, *p* < .001, η^2^_p_ = 0.11. The accuracy in the questions concerning items of misleading PEI was lower than in the questions about items only presented in the video, *t*(119) = 3.24, *p* = .004, Cohen’s *d* = 0.30, and questions about items shown in the video that were consistent with the news report, *t*(119) = 5.26, *p* < .001, Cohen’s *d* = 0.49. However, we found no differences between the questions about items only presented in the video and questions about items shown in the video that were consistent with the news report, *t*(119) = 2.12, *p* = .104, Cohen’s *d* = 0.19 (see Table 3).

The main effect of source credibility was not significant, *F*(1, 116) = 1.24, *p* = .267, η^2^_p_ = 0.01. For the purpose of source selection for our main study, we examined the misinformation effect size (i.e., the difference between the PEI absent and PEI present groups concerning the accuracy in the recognition test) for the high credibility source group (Misleading PEI Present: *M* = .77, *SD* = .11, 95% CI [.73, .80], Misleading PEI Absent: *M* = .83, *SD* = .10, 95% CI [.80, .87], *t*(58) = 2.57, *p* = .013, Cohen’s *d* = 0.66) and for the low credibility source group (Misleading PEI Present: *M* = .76, *SD* = .09, 95% CI [.72, .80], Misleading PEI Absent: *M* = .80, *SD* = .09, 95% CI [.77, .84], *t*(58) = 2.03, *p* = .047, Cohen’s *d* = 0.52).

We found a two-way interaction effect of the type of information addressed in the questions and misleading PEI on participants’ accuracy, *F*(1.866, 216.469) = 7.35, *p* = .001, η^2^_p_ = 0.06. To explore this interaction effect, we conducted three t-tests to compare the two groups of misleading PEI (present vs absent) for each type of question. Due to the Bonferroni correction, an alpha level of .0167 was used to interpret these t-tests to avoid type 1 error (Field, 2009). For the questions about the items of misleading PEI, participants who saw the news report with misleading PEI were less accurate (see Table 3) than participants who saw the news report that did not contain misleading PEI, *t*(118) = 4.18, *p* < .001, Cohen’s *d* = 0.76, 95% CI [0.39, 1.13]. As expected, there were no differences between the two groups of misleading PEI (present vs absent) concerning the accuracy in the questions about items only presented in the video, *t*(118) = 0.31, *p* = .759, Cohen’s *d* = 0.06, 95% CI [-0.30, 0.41], and the questions about items presented in the video that were consistent with the news report, *t*(118) = 1.99, *p* = .049, Cohen’s *d* = 0.36, 95% CI [0.002, 0.72]. Lastly, we found no two-way interaction between the type of information addressed in the questions and source credibility, *F*(1.866, 216.469) = 0.16, *p* = .839, η^2^_p_ = 0.001, misleading PEI and source credibility, *F*(1, 116) = 0.31, *p* = .578, η^2^_p_ = 0.003, and no three-way interaction between type of information addressed in the questions, misleading PEI and source credibility on participants’ accuracy, *F*(1.866, 216.469) = 0.03, *p* = .960, η^2^_p_ = 0.00.

[Table 3 here]

**Key Findings**

This pilot study examined the ability of two sources of misleading PEI to elicit a misinformation effect during a free recall and a multiple-choice recognition task. This was key to select the materials for our main study.

Regarding the exposure to misleading PEI, we found participants who read a news report (from social media or a credible journal) with misleading PEI reported/selected more units of misleading PEI than participants who saw a news report that did not contain misleading PEI. Similar results were found for both retrieval measures, showing the misinformation effect occurred in both tasks (free recall and multiple-choice recognition test). However, the results did not show a significant effect of credibility source on the number of misleading PEI recalled in the free recall task or accuracy in the multiple-choice recognition test. Considering our procedure effectively replicates the misinformation effect with both retrieval tasks, we adopted a similar procedure to generate the misinformation effect in our main study and selected the high credibility source of misleading PEI, which produced a stronger misinformation effect in both retrieval tasks.

**Table 1**

*Misleading PEI Items Presented in the News Report for the Misleading PEI groups*

| Item | Video | News Report |
| --- | --- | --- |
| Corpse | Men | Woman |
| Perpetrator’s hair colour | Brown | Blond |
| Weapon’s color | Black | Silver |
| Bag | Plastic | Cloth |
| Car trunk | Open | Closed |
| Corpse transported by | Two perpetrators | One of the perpetrators |
| Perpetrators agree to meet at | School | Coffee Shop |

**Table 2**

*Means, Standard Deviations, and 95% Confidence Intervals for the Number of Units of Misleading PEI Recalled According to Source Credibility and Misleading PEI*

|  | Source Credibility | | | | | |  | | |
| --- | --- | --- | --- | --- | --- | --- | --- | --- | --- |
|  | High | | | Low | | | Total | | |
| Misleading PEI | *M* | *SD* | *95% CI* | *M* | *SD* | *95% CI* | *M* | *SD* | *95% CI* |
| Present | 0.83 | 0.95 | [0.48, 1.19] | 0.90 | 1.42 | [0.37, 1.43] | 0.87 | 1.20 | [0.56, 1.18] |
| Absent | 0.30 | 0.54 | [0.10, 0.50] | 0.27 | 0.52 | [0.07, 0.46] | 0.28 | 0.52 | [0.15, 0.42] |
| Total | 0.57 | 0.81 | [0.36, 0.78] | 0.58 | 1.11 | [0.30, 0.87] | 0.58 | 0.97 | [0.40, 0.75] |

*Note.* PEI = Post-event Information.

**Table 3**

*Means, Standard Deviations, and 95% Confidence Intervals for the Accuracy in Recognition Test According to the Type of Information Addressed in the Questions and Misleading PEI*

|  | Type of Information Addressed in the Questions | | | | | | | | |  | | |
| --- | --- | --- | --- | --- | --- | --- | --- | --- | --- | --- | --- | --- |
|  | Video | | | Video and News Report | | | Misleading PEI | | | Total | | |
| Misleading PEI | *M* | *SD* | *95% CI* | *M* | *SD* | *95% CI* | *M* | *SD* | *95% CI* | *M* | *SD* | *95% CI* |
| Present | .80 | .15 | [.76, .84] | .82 | .13 | [.78, .85] | .67 | .20 | [.62, .72] | .76 | .10 | [.73, .79] |
| Absent | .79 | .15 | [.75, .83] | .86 | .12 | [.83, .89] | .81 | .15 | [.76, .84] | .82 | .09 | [.79, .84] |
| Total | .80 | .15 | [.77, .82] | .84 | .13 | [.81, .86] | .74 | .19 | [.70, .77] | .79 | .10 | [.77, .81] |

*Note.* PEI = Post-event Information.
