# Supplementary material for: The effect of response modality on witness statements when using the self-administered interview
Source: Psychiatr Psychol Law. 2024 Apr 7;32(3):337–53. doi: 10.1080/13218719.2024.2313977 (PMC12123897; doi:10.1080/13218719.2024.2313977)
Supplement: Supplemental Material [file TPPL_A_2313977_SM3017.docx]

**Supplementary Materials**

Free Recall Task Instructions

**Explaining the Purpose of the Interview**

First, the interviewer explained the general purpose of the interview: *My goal is to help you to remember as much information as possible about the video you watched a week ago. I will ask you to tell me everything you remember about the video, with as much detail as possible.*

**Report Everything, Asking Not to Guess, and Transferring Control**

An object was used for exemplifying the level of detail the interviewee was expected to provide in his account. The interviewer asked participants to describe an object (e.g., interviewer's watch) and then comprehensively described that same object while adding minor details concerning such object that interviewees left out in their previous description (e.g., detailed description of the clock hands). The interviewer proceeded with explaining the report everything mnemonic: *Just like I exemplified with this object, when you are reporting what you remember about the crime, please tell me everything you remember with as much detail as you can. Please tell me everything you remember, even the details that may seem irrelevant to you, are very important to me. Some people omit information they believe are not important. However, I am interested in you to tell me everything that pops into your mind.*

Participants were then asked not to guess and control of the interview was transferred to the interviewee: *Don’t panic if you can’t remember something. Just say that you do not remember rather than trying to guess or assume information. You have all the time you need, so don’t worry about taking too long when thinking about the video or providing information. You can take a break whenever you like, and we can stop for a few minutes at any time. Although I have been leading the interview so far, I will stop in a moment. I will ask you to tell me everything you remember about the video, in whatever order you wish, without interruptions. I will be here to help you whenever you need. Is this all right with you?*

**Mental Reinstatement of Context**

Mental reinstatement of context (with eye-closure) was applied and maintained throughout retrieval: *Now I would like you to close your eyes and picture the crime scene of the video. Keep your eyes shut throughout the entire interview as it aids concentration and can help you form a clearer image of the crime in your mind. Please close your eyes (…) Try to remember the day you watched the video (…) Think about what you were doing that day (…) Think about how you were feeling that day (…) Now focus on the crime you’ve watched that day and try to get a clear picture of the crime scene in your mind (…) as clear as possible (…) think of what persons were there (…) think of all actions that occurred (…) how everything was located at the crime-scene (…) all the object and environmental details of the scene (…) think of all the sounds and conversations you heard in the scene (…) and when you’re ready and you have a clear picture of the crime scene in your mind please focus on what happened and tell me everything you remember in any order you want and at the pace you desire. Please keep your eyes closed throughout.*

**Closure**

After finishing their recall attempt, all participants were asked if there was anything else they wished to add to their reports: *Ok, is there anything else you can remember?* Lastly, appreciation for participants’ hard work and cooperation was acknowledged: *Thank you very much for your cooperation and time, it is very much appreciated.*
